# The effect and safety of exercise therapy in patients with systemic sclerosis: a systematic review

**DOI:** 10.1093/rap/rkz044

**Published:** 2019-12-09

**Authors:** Sophie I E Liem, Theodora P M Vliet Vlieland, Jan W Schoones, Jeska K de Vries-Bouwstra

**Affiliations:** 1 Department of Rheumatology, Leiden, The Netherlands; 2 Department of Orthopaedics, Rehabilitation Medicine and Physical Therapy, Leiden, The Netherlands; 3 Walaeus Library, Leiden University Medical Center, Leiden, The Netherlands

**Keywords:** systemic sclerosis, scleroderma, exercise therapy, exercise, review

## Abstract

Given the shortcomings of previous literature reviews evaluating the effect and safety of exercise therapy in SSc, we aimed to carry out a systematic review of the literature specifically on this topic. A structured search strategy was performed in Medline (via PubMed) and other electronic databases from 1990 to 3 September 2019. Randomized controlled trials, observational designs, conference abstracts and trial registrations were included if they concerned SSc patients ≥18 years of age, exercise therapy and reported outcomes related to physical functioning. Nine articles were included. Four randomized controlled trials compared (a) hand exercises, (b) orofacial exercises, (c) aerobic exercises or (d) aerobic exercises plus resistance training with no exercise, demonstrating effects on hand function (a), maximum mouth opening (b), peak oxygen uptake (c + d) and quality of life. All five observational studies concerning hand, orofacial, aerobic and/or strengthening exercises reported improvements of hand function, mouth opening, aerobic capacity and/or muscle strength. In conclusion, the evidence on the effect and safety of exercise therapy in SSc is scanty.

**Key messages**

- Literature on the effectiveness and safety of exercise in patients with SSc is scarce.
- Beneficial effects of hand, orofacial or aerobic exercises are reported in patients with SSc.
- Well-designed trials are needed to determine the optimal timing, content and dosage of exercise in SSc.

## Introduction

SSc is a rare, systemic autoimmune disease characterized by skin fibrosis and vasculopathy [1]. In addition to the skin, multiple organ systems, including the musculoskeletal, cardiac, pulmonary and gastrointestinal systems, are involved, resulting in a broad range of symptoms [2].

For various reasons, including joint pain and limited range of motion, fatigue and dyspnoea as a result of lung involvement, the exercise capacity of SSc patients was found to be limited in comparison to healthy controls [3, 4]. Moreover, apart from impairments in activities and participation, their level of physical activity in daily life appears to be relatively low [5]. To overcome or decrease these impairments and limitations, aside from medical treatment, the usage of rehabilitation interventions is advocated in the management of patients with SSc [6]. Exercise therapy is an important element of rehabilitation, aiming to improve the overall functioning of the individual and to support patients to meet the demands of daily living [7]. Indeed, the usage of physical therapy in patients with SSc is substantial, with 50–60% of patients visiting a physical therapist over a period of 12 months [8, 9]. Despite this relatively wide use, the evidence for the effect of exercise therapy appears to be scanty.

To the best of our knowledge, to date three literature reviews, one of which was performed systematically, have assessed the effect of exercise therapy in patients with SSc. A systematic review by Willems *et al.* [10] published in 2015 assessed the effect of various non-pharmacological interventions in SSc, including exercise therapy. That review included three studies specifically on exercise therapy [11–13], two pertaining to orofacial exercises [11, 13] and one to muscle-strengthening and aerobic exercises [12]. Of these three studies, only one was a randomized controlled trial (RCT) and met the criteria for high methodological quality [13]. The systematic review also included eight studies on multidisciplinary interventions, of which exercise therapy formed a part [14–21]. However, given that the programmes were comprehensive, the effect of exercise therapy alone cannot be determined from these studies. A more recent literature review specifically addressed aerobic and resistance exercise in SSc, with interventions categorized into aerobic exercises and aerobic exercises in combination with resistance exercise, and patients divided into those with and without pulmonary involvement [22]. This review included 10 studies [12, 15, 16, 19, 23–28], four being RCTs. One RCT concerned the evaluation of an intervention combining a personalized physical therapy programme with occupational therapy, meaning that the effect of physical therapy alone cannot be determined [28]. Although this literature review comprised one more recent study [28], studies with smaller study populations [23, 27] and case reports [24–26], it was not performed systematically. The authors concluded that SSc patients who participate in exercise programmes involving aerobic exercise and aerobic exercise combined with resistance training improve in exercise tolerance, cardiorespiratory fitness, walking distance, muscle strength and function, in addition to health-related quality of life [22]. The most recent review, published in 2019 by Mugii *et al.* [29], was not performed systematically and included 11 studies concerning rehabilitation therapy applied for SSc categorized into four domains: hand, face, global and pulmonary rehabilitation. Three studies [11, 13, 27] concerned exercise therapy specifically, and eight studies concerned comprehensive care [14, 15, 17, 18, 28, 30–32]. Of these studies, one study was not yet mentioned in the two other reviews [32]. That review concluded that although few high-quality RCTs have been conducted to date, previous studies indicated the effect of rehabilitation therapy for decreasing local and systemic disabilities, resulting in improved quality of life.

In conclusion, a systematic literature review specifically addressing the effect of exercise therapy and distinguishing different types of exercise programmes (hand exercises, orofacial exercises and aerobic and muscle-strengthening exercises) is lacking. A comprehensive overview of the already existing evidence and ongoing research is needed to identify the gaps in knowledge, to plan future research projects and to develop specific guidelines for physical therapists. For this purpose, the aim of the present study was to identify and summarize the literature on the effect and safety of exercise therapy in patients with SSc and provide an overview of ongoing research in this field.

## Methods

This systematic review was conducted according to the PRISMA guidelines [33]. Moreover, we adhered largely to the AMSTAR 2 tool [34], with the exception of the protocol registration before commencement. This is a drawback of our approach.

### Search strategy

The databases Medline (via PubMed), Embase, Web of Science, Cochrane Library, PsycINFO, CENTRAL, Emcare, Academic Search Premier, ScienceDirect and Wiley Online Library were searched from 1990 to 3 September 2019. To compose the search strategy, we used PICO (Patient, Intervention, Comparison, Outcome) research question: ‘What is the effect of exercise therapy (I) on outcomes regarding physical functioning (O) in systemic sclerosis patients (P), if possible in comparison (C) to a different type of exercise, no intervention or a non-exercise intervention? The broad computerized search strategy consisted of the combination of two main components (for the detailed search strategy, see Supplementary Material, Search Strategy section, available at *Rheumatology Advances in Practice* online): SSc and exercise therapy. The search strategy was formulated with the help of a trained librarian (J.W.S.). Additionally, the reference lists of relevant articles were hand searched for additional relevant studies.

### Inclusion criteria and study selection

Studies were eligible for inclusion in the review if the study was published in English or Dutch, because these languages are mastered by the authors; the publication date was between 1990 and 3 September 2019; the study included participants ≥ 18 years of age with SSc taking part in an exercise programme; and the intervention consisted only of exercise therapy reported on one or more outcomes regarding physical functioning. Moreover, we included RCT, controlled clinical trials and observational designs. Over the past years, non-pharmacological treatment, and thus exercise therapy, of SSc has gained increasing attention. Therefore, we screened conference abstracts to check for potentially missed studies for the literature synthesis. Moreover, both conference abstracts and clinical trial registrations were searched to make an inventory of ongoing and upcoming projects in this field. All inclusion criteria were applicable, except for the criterion that it should include results/data.

For the purpose of this review, exercise therapy was defined as planned, repetitive movements, which were, at least in part, supervised by a physical therapist [35]. All studies on exercises fulfilling these criteria were accepted, irrespective of the type, frequency, intensity, mode or duration. Excluded were studies on exercise therapy that were only provided unsupervised or where exercise therapy was part of a larger multidisciplinary intervention.

The procedure for the selection of the studies was based on the recommendations of Furlan *et al.* [36]. Initially, two reviewers independently screened titles and abstracts with the inclusion criteria in mind. Titles and abstracts that passed this screening underwent a full-text review using the complete set of inclusion and exclusion criteria, performed independently by the same two reviewers. In the eventof discrepancies in agreement, abstracts or full-text articles were reviewed by a third investigator (J.K.V.-B.).

### Quality assessment

The methodological quality of both randomized and non-randomized studies was evaluated using the Downs and Black checklist, which consists of 27 criteria representing five domains: study reporting, external validity, internal validity-bias, internal validity-confounding and power [37]. The Cochrane Musculoskeletal Group has identified the Downs and Black checklist as one of six useful tools to assess risk of bias, including bias in non-randomized (observational) studies [38]. Item 27 of the checklist regarding the domain power was modified slightly. The original question of item 27 (‘Did the study have sufficient power to detect a clinically important effect where the probability value for a difference being due to chance is less than 5%?’) was changed to ‘Did the study include a power analysis?’, with answer options yes or no (Supplementary Material, Modified Downs and Black Checklist for evaluating study quality section, available at *Rheumatology Advances in Practice* online). For each study, two reviewers (S.I.E.L. and T.P.M.V.V.) independently scored all 27 items, and disagreements were resolved by consulting a third reviewer (J.K.V.-B.). A total quality score was then calculated by adding up all item scores (zero or one, except for item 5, where 0 = no, 1 = partially, and 2 = yes), resulting in a maximum possible score of 28. The checklist does not provide a cut-off point for determining a high-quality study. Therefore, we arbitrarily defined that a study was considered high quality with a score higher than two-thirds of the maximum possible score (if all 28 items were applicable, then a score of ≥ 19 was considered as high quality).

### Data extraction

Data extraction concerned general characteristics of the study, patient characteristics, intervention characteristics, outcome measurements and results.

General characteristics of the studies included publication year, study design, country and authors. Patient characteristics included the inclusion and exclusion criteria, the number of participants, mean age (in years), mean disease duration (in years) and female/male ratio. It was also recorded if, and how, the diagnosis of SSc was established (based on published criteria, clinical diagnosis, or not reported) [39, 40].

Regarding the intervention, the type of exercises, the frequency, intensity, mode and duration were recorded. Exercise programmes were categorized into three groups: hand exercises; orofacial exercises; and muscle-strengthening and aerobic exercises.

The primary outcomes of interest to this review were measurements of physical functioning. For that purpose, we considered measures of general physical functioning, such as the HAQ or Short Form 36 (SF-36) or 12 physical component scale. In addition, for every category of exercises (hand, orofacial, aerobic and/or muscle-strengthening exercises), we used a list of probable outcomes of physical functioning of particular interest for that type of exercise. For hand exercises, these were measures of hand function or grip strength. For orofacial exercises, we considered oral aperture. For aerobic and/or muscle-strengthening exercises, 6 min walking test, peak oxygen uptake, oxygen saturation, heart rate, metabolic equivalent, repetition and muscular threshold were included. In the case of doubt about whether an outcome measure could be considered to be a measure of physical functioning, the authors discussed it together for consensus. Additionally, adherence rates and adverse events were recorded if reported.

One reviewer (S.I.E.L.) extracted the data and entered data items into an electronic day entry programme (Microsoft Excel), with all data being cross-checked by one of the authors (T.P.M.V.V.).

### Analysis

Owing to the heterogeneity of the included studies, in particular with regard to the study designs and contents of the intervention, a meta-analysis was not performed. Therefore, a descriptive analysis was used to assess the effect of exercise therapy.

## Results

### Search results

**Fig. 1:**
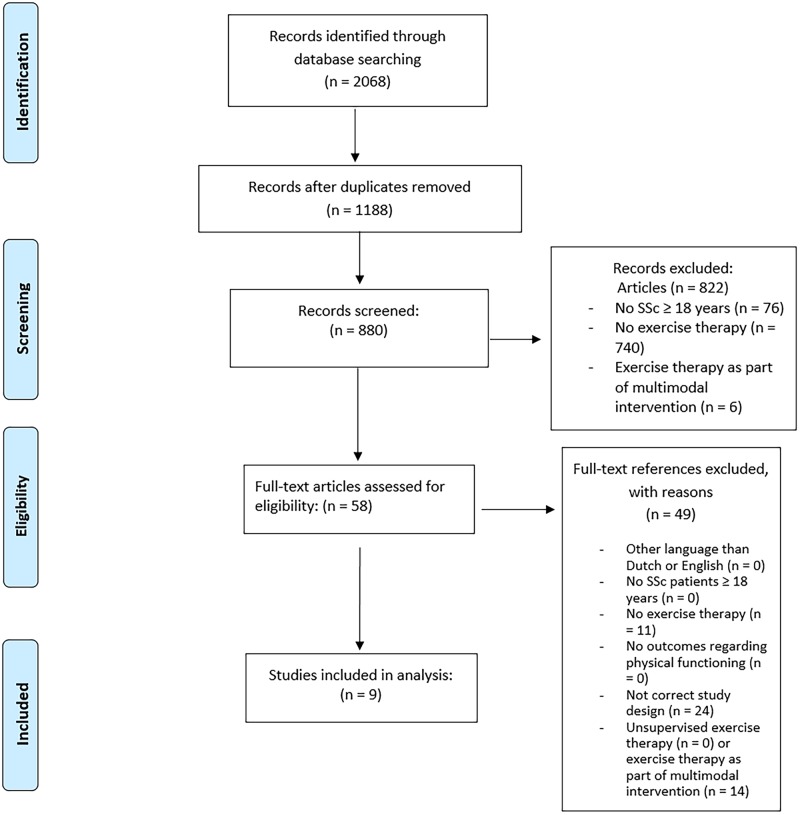
Flow diagram of selection process

The systematic search yielded 880 unique article references, of which 58 were selected for full-text review (Fig. 1). Nine articles met the inclusion criteria. No additional titles were obtained by checking the references of included papers and the available literature reviews [10, 22, 29].

### Characteristics of the included studies

The characteristics of the included studies are shown in Table 1. The total number of participants included in the various studies varied from 4 to 48. The average age of the patients ranged from 44.0 to 69.6 years, whereas the mean disease duration ranged from 3.5 to 12.6 years and the percentage of women ranged from 75 to 100%. The diagnosis of SSc was based on the ACR and EULAR criteria 2013 [40] in three studies [41–43], on the preliminary criteria for the classification of SSc of the ARA criteria 1980 [39] in four studies [12, 13, 23, 44] and not reported in two studies [11, 27].

**Table 1:** Main characteristics of included studies

First author, year, country [reference]	Study design	Subjects	Intervention	Type of supervision	Primary outcomes
**Hand exercises**					
Piga, 2014, Italy [44]	RCT	E: *n* = 10, mean age=57.0 years, mean disease duration = 6.9 years, female *n* = 10, diffuse/limited subtype 2:8 C: *n* = 10, mean age = 57.4 years, mean disease duration = 6.7 years, female *n* = 10, diffuse/limited subtype 2:8	Strengthening and mobility hand exercises done at home using the Re.Mo.Te device; 50 min, 5 days/week for 12 weeks C: exercises using common daily-life objects	Every patient received individual 1 h training on the use of the device, along with an illustrated booklet describing the exercises. Every workout was remotely monitored by physicians through the telemonitoring interface	Hand function measured by HAQ, functional index for hand OA and the hand mobility in scleroderma test
Landim, 2017, Brazil [41]	OD	*n* = 22, mean age = 48.09 years, mean disease duration = 11.19 years, female *n* = 18, diffuse/limited subtype 6:16	Home-based self-management programme consisting of hand exercises and concise instructions about SSc	Instructions in the program	Hand pain (visual analog scale) and hand function (Cochin hand function scale)

**Orofacial exercises**					
Yuen, 2011, USA [13]	RCT	E: *n* = 13, mean age = 51.8 years, mean disease duration = 11.3 years, female *N* = 11, diffuse/limited subtype 8:5, mean oral aperture = 27.4 mm C: *n*= 15, mean age = 50.9 years, mean disease duration = 6.2 years, female *n* = 11, mean, diffuse/limited subtype 6:9, mean oral aperture = 32.4 mm	Manual mouth-stretching and oral-augmentation exercises; 6 min, twice daily for 26 weeks C: no exercise	Patients were taught to perform manual mouth-stretching and oral augmentation exercises by a trained research coordinator. Handouts with pictures showing the exercises were given	Oral aperture
Pizzo, 2003, Italy [11]	OD	*n* = 10, mean age = 56.8 years, female *n* = 10, mean maximal mouth opening = 26 mm	Mouth-stretching exercises and oral augmentation exercises, 20 minutes, twice daily for 18 weeks	Patients were instructed by one of the investigators to perform the exercise programme	Maximal mouth opening

**Aerobic and muscle-strengthening exercises**					
Mitropoulos, 2018, UK [42]	RCT	E1: *n* = 11, mean age = 69.1 years, mean disease duration = 7.8 years, limited subtype E2: *n* = 11, mean age = 65.1 years, mean disease duration = 7.7 years, limited subtype C: *N* = 12, mean age = 62.2 years, mean disease duration = 6.3 years, limited subtype	E: 2 days/week for 12 weeks, 30 min of 30 s high-intensity-interval training followed by 30 s passive recovery. In the E1 group this was performed on an arm-crank ergometer and in the E2 group on a cycle ergometer C: no exercise	Supervised sessions at sport venues of the hospital	VO_2_peak EQ-5D-5-L 6 min walking test^a^
Mitropoulos, 2019, UK [43]	RCT	E: *n* = 16, mean age = 69.6 years, mean disease duration = 8 years, limited subtype C: *n* = 16, mean age=63.6 years, mean disease duration=8 years, limited subtype	E: exercise programme consisting of: (a) high-intensity interval training; and (b) resistance training (five upper body exercises in a circuit row for three circles interspersed by 2–3 min), 2 days/week for 12 weeks C: no exercise	Supervised sessions at sport venues of the hospital	VO_2_peak^a^
Oliveira, 2009, Brazil [23]	OD	E: *n* = 7, mean age = 45.6 years, mean disease duration = 12.6 years, female *n* = 7, diffuse/limited subtype 2: 5, mean forced vital capacity for diffuse subtype = 77.5% of predicted and for limited subtype 103.8% of predicted	Aerobic exercise (30 min of treadmill walking at moderate intensity), 40 min, 2 days/week for 8 weeks	Supervised sessions	VO_2_peak, oxygen saturation^a^
Pinto, 2011, Brazil [12]	OD	*n* = 11, mean age = 44.0 years, mean disease duration = 7.36 years, female *n* = 11, diffuse/limited subtype 8:3	Aerobic training (20 min of treadmill exercise at a heart rate of ∼70% of VO_2_peak), resistance training (30 min, four sets of 8–12 maximal repetitions for the main muscle groups), 2 days/week for 12 weeks	Supervised training	Oxygen uptake, highest exercise load for bench and leg press^a^
Alexanderson, 2014, Sweden [27]	Single subject experimental design	*n* = 4, mean age = 66.5 years, mean disease duration = 3.5 years, female *N* = 3, lung fibrosis with 50–80% forced vital capacity/100% forced vital capacity = 2:2	Aerobic exercise (ergometer cycling of maximum 30 min. Intensity increased from light exertion to 15 on a Borg scale) and muscular endurance training for shoulder and hip flexors, 30–50 min, 3 days/week for 8 weeks	Supervised by a trained physical therapist	6 min walking test

a For these studies the primary outcomes were not defined in the text, but we chose the main outcomes based on our definition of physical functioning outcomes in the text.

C: control group; E: experimental group; OD: observational design; RCT: randomized controlled trial; VO_2_peak: peak oxygen consumption.

### Methodological quality

The methodological quality of the included studies is shown in Table 2. Of the nine included studies, one was rated as being of high quality [13]. There was an agreement >80% of the scores on the individual items. In case of discordance, consensus was achieved after discussion with the third reviewer.

**Table 2:** Methodological quality of the included studies

First author, publication year [reference]	Study reporting	External validity	Internal validity, bias	Internal validity, confounding	Poweranalysis	**Quality score** ^a^	Level of quality	Not applicable
**Hand exercises**								
Piga, 2014 [44]	1, 2, 3, 4, 5, 6, 7, 9, 10		16, 17, 18, 19, 20	21, 22, 23		17/28	Low	
Landim, 2017 [41]	1, 2, 3, 4, 6, 7, 8, 10	13	18, 20	25		12/25	Low	17, 22, 23

**Orofacial exercises**								
Yuen, 2011 [13]	1, 2, 3, 4, 5 (2x), 6, 7, 9, 10	13	15, 16, 17, 18, 19, 20	21, 23, 25, 26		21/28	High	
Pizzo, 2003 [11]	1, 2, 4, 6, 7, 8, 9, 10		16, 19, 20	21, 26		13/26	Low	17, 22

**Aerobic and muscle-stregthening exercises**								
Mitropoulos, 2018 [42]	1, 2, 3, 4, 5, 6, 7, 9, 10	13	16, 17, 18, 19, 20	21, 23, 26		18/28	Low	
Mitropoulos, 2019 [43]	1, 2, 3, 4, 5, 7, 9, 10	13	16, 17, 18, 19, 20	21, 23, 26		17/28	Low	
Oliveira, 2009 [23]	1, 2, 3, 4, 6, 7, 10	13	16, 18, 19, 20			12/25	Low	17, 22, 23
Pinto, 2011 [12]	1, 2, 3, 4, 6, 7, 9, 10	13	18, 20	26		12/23	Low	5, 17, 22, 23
Alexanderson, 2014 [27]	1, 2, 3, 4, 6, 7, 9	13	16, 18, 19	26		12/24	Low	17, 22, 23, 25

Only the numbers for fulfilled criteria are reported.

a Quality score is the sum of positive scores. Studies are considered of high quality when their total quality score reflects at least two-thirds of answered items.

### Effect of exercise therapy

The effect of exercise therapy per study is shown in Table 3.

**Table 3:** Results of exercise interventions of included studies

First author, year, country [reference]	Primary outcomes at baseline	Adherence, %	Results
**Hand exercises**			
Piga, 2014, Italy [44]	E: HAQ: 1.49 Dreiser’s index: 13.9 HAMIS right hand: 5.2 HAMIS left hand: 4.7 C: HAQ: 1.56 Dreiser’s index: 14.0 HAMIS right hand: 4.7 HAMIS left Hand: 2.2	93.4 (range 71.4–98.8)	The experimental group showed significant improvements in Dreiser’s index (13.9–7.7), HAQ (1.49–0.81) and the HAMIS (right hand: 5.2–3.3; left hand: 4.7–2.2) over time, but differences between groups were not significant (change over time in control group for Dreiser’s index: 14.0–9.50; HAQ: 1.56–1.09; HAMIS right hand: 4.7–3.2; HAMIS left hand: 2.2–1.7).
Landim, 2017, Brazil [41]	Pain visual analog scale: 3.97 Cochin hand function scale: 19.24	Not determinable	Significant improvements in hand pain measured by visual analog scale (3.97 *vs* 2.21, *P* = 0.0022), Cochin hand function scale (19.24 *vs* 12.48, scleroderma HAQ (0.95 *vs* 0.48 and handgrip strength improved (14.43 *vs* 19.00)

**Orofacial exercises**			
Yuen, 2011, USA [13]	Oral aperture (mm) E: 27.4 C: 32.4 *P* = 0.049 between groups	48.9 (s.d. = 32.6)	In 3 months, the experimental group showed a significantly larger change (i.e. increase) in the size of oral aperture compared with the control group (2.81 *vs* −0.61 mm). This effect did not last at the 6-month evaluation (2.75 *vs* 2.33 mm). There was a significant difference in the overall change of the oral aperture size in the orofacial exercise group (2.75 mm) but not the no-exercise group (2.33 mm)
Pizzo, 2003, Italy [11]	Maximal mouth opening (mm): 26	100	The maximum mouth opening improved significantly from 26 to 36.7 mm after the intervention

**Aerobic and muscle-strengthening exercises**			
Mitropoulos, 2018, United Kingdom [42]	VO_2_peak (ml/kg/min): E1: 17.7 E2: 14.6 C: 14.3	E1 (arm crank): 92 E2 (cycle ergometry): 88	In both intervention groups, values of VO_2_peak were greater post-exercise intervention compared with the control group (significantly for the arm crank group). Both intervention groups reported improved quality of life
Mitropoulos, 2019, United Kingdom [43]	VO_2_peak (ml/kg/min): E: 20.6 C: 15.7	Not determinable	VO_2_peak was significantly greater in the exercise group (25.6±7.2 ml/kg/min) compared with the control group after the exercise intervention
Oliveira, 2009, Brazil [23]	VO_2_peak (ml/kg/min): 19.72 Metabolic equivalent: 5.63	100	Significant improvement in VO_2_peak (19.72 *vs* 22.27), peak exercise oxygen saturation (84.14 *vs* 90.29) and metabolic equivalent 95.63–6.36)
Pinto, 2011, Brazil [12]	Highest exercise load of leg press: 67 kg; and bench press 47 kg VO_2_peak: 21.6 ml/kg/min	Not determinable	Significant improvement in muscle strength and function, time to exhaustion, heart rate at rest, and the workload and time of exercise at ventilatory thresholds and peak of exercise
Alexanderson, 2014, Sweden [27]	6 min walk test at baseline unknown	98	No patient showed a statically significant change in physical walking distance during the 6 min walk test. Three patients significantly improved with respect to muscular endurance concerning hip and shoulder flexion. Aerobic capacity measured by treadmill test improved in one patient statistically significant and clinically significant in one patient. Reduced fatigue measured by visual analog scale in three patients.

C = control group; E: experimental group; HAMIS: HAnd Mobility in Scleroderma; VO_2_peak: peak oxygen consumption.

### Hand exercises

Regarding hand exercises, two studies were identified [41, 44], with both showing beneficial effects on hand function. Piga *et al.* [44] conducted a study in which an exercise programme consisting of strengthening and mobility exercises supported by telemedicine was compared with a similar intervention with the aid of common daily-life objects in 20 patients, with 10 patients in each group. SSc patients showed an improvement of hand function measured by the Functional Index for Hand OsteoArthritis (FIHOA, from 13.9 to 7.7 in the intervention group *vs* from 14.0 to 9.50 in the control group, *P* < 0.01), but the between-subject effect was not significant (*P* = 0.496). The HAQ (1.49 to 0.81, *P* = 0.016), and the HAnd Mobility in Scleroderma (HAMIS, right hand: from 5.2 to 3.3, *P* = 0.016; left hand: from 4.7 to 2.2, *P* = 0.075) improved significantly only in the telemedicine group. Patients in the experimental group performed 93.4% (range 71.4–98.8%) of the scheduled exercise sessions, and no adverse events were recorded.

Landim *et al.* [41] evaluated the effect of a home-based hand care programme in 22 SSc patients. The primary outcomes, hand pain measured by visual analog scale and the Cochin hand function scale, both improved significantly (hand pain: 3.97 *vs* 2.21, *P* = 0.0022; Cochin hand function scale: 19.24 *vs* 12.48, *P* < 0.0001). Furthermore, significant improvements were noted in secondary outcomes, such as the scleroderma HAQ (0.95 *vs* 0.48, *P* < 0.0001) and grip strength (14.43 *vs* 19, *P* = 0.0022). Patients considered the programme easy to follow, and no adverse effects related to exercises were noted.

### Orofacial exercises

Two studies [11, 13] reported the effects of orofacial exercises on the maximal oral aperture. In the RCT of Yuen *et al.* [13], 48 patients were included for a multifaceted oral health intervention. Of those 48 participants, 28 participants, with a mean oral aperture of <40 mm at baseline, were additionally randomized between orofacial exercise instructions (*n* = 13) and no exercises (*n* = 15). In a subgroup analysis including only the patients with an oral aperture <40 mm at baseline, this study demonstrated a significantly larger increase in oral aperture at 3 months for the intervention group compared with those receiving no exercise (2.81 *vs* −0.61 mm, *P* = 0.01). However, this effect did not last at the 6-month evaluation (2.75 *vs* 2.33 mm, *P* = 0.19). In an observational study in 10 patients with SSc and a maximal mouth opening ≤ 30 mm, Pizzo *et al.* [11] showed a mean improvement of oral aperture of 10.7 ± 2.06 mm after the exercise programme (*P* < 0.0049).

The adherence rates to the orofacial exercise were 48.9% [13] and 100% [11]. Reasons for discontinuation were soreness of the lips or at the jaw point, decreased pigmentation at the mouth corner, forgetfulness or not having the time [13]. It was stated that, with the exception of transient muscular fatigue, no adverse effects were reported in one study [11], whereas the other did not mention adverse effects [13].

### Aerobic and muscle-strengthening exercises

Five studies evaluated the effect of aerobic exercise programmes in SSc patients, of which two programmes included only aerobic training [23, 42] and three programmes combined aerobic exercise and resistance training [12, 27, 43].

The two most recent studies, both RCTs, on aerobic exercise and aerobic exercise in combination with muscle-strengthening exercises, were performed by Mitropoulos *et al.* [42, 43, 45] The first RCT [42] compared three groups: two with high-intensity interval training (arm cranking and cycling) and one control group (no exercise). The exercise groups underwent a 12-week supervised exercise programme. Twice weekly, they performed 30 s high-intensity interval training interspersed with 30 s of passive recovery for a total of 30 min. Peak oxygen uptake increased in both exercise groups significantly post-intervention compared with baseline. Quality of life was assessed as a secondary outcome and improved significantly in both exercise groups [42].

In the second, most recent RCT [43] 32 patients with limited SSc were randomly allocated to an exercise group (*n* = 16) or a no-exercise group (*n* = 16). Similar high-intensity training to that in the other study from the same authors [42] was combined with resistance training comprising five upper-body exercises (chest press, arms lateral raise, biceps curl, triceps extension and handgrip dynamometer). This exercise programme also lasted 12 weeks and was performed twice weekly. Peak oxygen uptake and transcutaneous oxygen pressure of the exercise group improved significantly when compared with the control group after the intervention [43].

Oliveira *et al.* [23] noted a significant improvement in peak oxygen consumption (19.72 *vs* 22.27, *P* = 0.006) after an 8-week programme consisting of moderate-intensity aerobic exercise on a treadmill in seven SSc patients. Furthermore, peak blood lactate significantly decreased (1.43 *vs* 1.84, *P* = 0.01). Peak exercise oxygen saturation significantly improved in comparison to baseline (84.14 *vs* 90.29, *P* = 0.048), whereas resting oxygen saturation did not improve after exercise.

In the study by Pinto *et al.* [12], 11 SSc patients participated in a 12-week combined resistance and aerobic training programme, with twice weekly sessions. Patients significantly improved with respect to muscle strength and function, time to exhaustion, heart rate in resting conditions and, in addition, the workload and time of exercise at ventilatory thresholds and peak of exercise were increased.

Using a single-subject experimental design, Alexanderson *et al.* [27] enrolled four SSc patients (three women and one man) in an 8-week exercise programme consisting of aerobic exercise corresponding to 15 on the Borg rating of perceived exertion scale (strenuous) and muscular endurance training, three times per week. Three patients improved significantly with respect to muscular endurance concerning hip and shoulder flexion. Aerobic capacity measured by treadmill test improved significantly in one patient, and clinical aerobic capacity improved in another patient.

### Safety of exercise therapy

As shown in Table 4, in seven of the nine studies no adverse events related to the exercise programmes were reported [12, 23, 27, 41–44]. Pizzo *et al.* [11] reported transient muscular fatigue as an adverse event. Although the study by Yuen *et al.* [13] did not report adverse events in the manuscript, the reasons for discontinuation for both groups were specified. These included sickness, diagnosis of cancer, incarceration, complaint of sore throat after dental cleaning in the intervention group; and hip replacement, military service and unable to re-schedule the final visit before termination of the study in the control group. Moreover, reasons for discontinuation in the studies of hand exercises were major abdominal surgery [44] and transport problems [41] in the exercise groups. In the study by Mitropoulos *et al.* (2018) [42] and in the study by Oliveira *et al.* [23], two dropouts in the intervention group were recorded, but the reasons were not specified. The studies by Pizzo *et al.*, Pinto *et* *al.*, Alexanderson *et al.* and Mitropoulos *et al.* (2019), all included patients completed the follow-up [11, 12, 27, 43].

**Table 4:** Safety of exercise therapy

First author, publication year [reference]	Adverse events	Dropouts/protocol violations
**Hand exercises**		
Piga, 2014 [44]	None reported	E: one patient discontinued the exercise protocol because of major abdominal surgery and was withdrawn from the trial C: two patients reported discontinuing the protocol for >1 week for no specific reason and were withdrawn from the study
Landim, 2017 [41]	None reported	Five patients did not return for re-evaluations and were excluded. In the flow diagram, transportation problems are given as the reason

**Orofacial exercises**		
Yuen, 2011 [13]	Not determinable	E: four participants dropped out. Some of the known reasons for participant dropout included sickness, diagnosis of cancer, incarceration, and complaint of sore throat after dental cleaning C: five participants dropped out. Some of the known reasons for this included hip replacement, military service, and unable to re-schedule the final visit before termination of the study
Pizzo, 2003 [11]	Mid-muscular fatigue at the cheek and the temporomandibular joint was reported in 10/10 and 4/10 subjects, respectively. This occurred during the exercise programme and disappeared within 30 min after finishing the exercises	No dropouts

**Aerobic and muscle-strengthening exercises**		
Mitropoulos, 2018 [42]	None reported	One dropout for each exercise group. Reasons are not specified
Mitropoulos, 2019 [43]	None reported	No dropouts
Oliveira, 2009 [23]	None reported	Nine patients agreed to participate and seven completed the study. Reasons are not given
Pinto, 2011 [12]	None reported	No dropouts
Alexanderson, 2014 [27]	None reported	One participant missed two of in total 24 exercise sessions owing to medical investigations of increased lung symptoms

C: control group; E: experimental group.

### Conference abstracts and clinical trial registrations

A total of 631 conference abstracts and clinical trial registrations have been reviewed independently by S.I.E.L. and T.P.M.V.V. None of the conference abstracts could be linked to a published paper that we had missed. Two conference abstracts and one clinical trial registration pointed out ongoing research (Table 5).

**Table 5:** Overview of ongoing and upcoming projects concerning exercise therapy in SSc

First author, country [reference]	Study design	Subjects	Intervention	Outcomes
**Hand exercises**				
Kwakkenbos, multicentre [48, 49]	RCT	586 SSc patients with at least mild hand function limitations (Cochin hand function scale ≥3)	E: online hand-exercise intervention, 3 months C: usual care	Cochin hand function scale

**Orofacial exercises**				
Sydow, Belgium, [46, 47]	OD	SSc patients with maximal oral aperture <40 mm	E1: exercises with jaw motion device E2: mouth-stretching exercises In both groups, patients had to exercise for 10 min, three times/day for 3 months	Mouth opening

**Aerobic and muscle-strengthening exercises**				
Ferrari, Italy [50]	Single-blind RCT, parallel assignment	33 SSc patients	E: home-based exercise programme consisting of aerobic exercise on a stationary bicycle, muscle-endurance training of upper limb and stretching exercises for finger joint motion. C: encouragement to perform generic aerobic physical activity	6 min walking test, maximum oxygen consumption, handgrip strength, one repetition maximum of biceps strength, muscular strength of lower limbs, hand mobility in scleroderma test

C: control group; E: experimental group; OD: observational design; RCT: randomized controlled trial.

Preliminary results of an exploratory study on the effects of orofacial exercises in SSc patients have been published as a conference abstract. This study assessed two different exercise approaches designed to increase oral aperture. The first group exercised with a passive jaw motion device and the second group did mouth-stretching exercises. Both groups had to exercise for 10 min, three times per day for 3 months. Outcome measures were oral aperture and compliance of the intervention [46, 47].

The Scleroderma Patient-centered Intervention Network has an ongoing RCT that will evaluate the effect of an online hand-exercise intervention study, in addition to usual care, on hand function and health-related quality of life in SSc patients with at least mild hand function limitations [48–50].

Moreover, a randomized, controlled 6-month parallel group study is registered, wherein patients will be randomly assigned to the home care rehabilitation group or the control group. The intervention consists of a physical exercise programme at home: aerobic exercise on a stationary bicycle, muscular endurance training of the upper limb three times a week and daily stretching exercises for finger joint motion. The control group will be given generic recommendations to increase physical activity. This RCT has completed its recruitment, but no results are published yet [51].

## Discussion

This systematic review on the effect of exercise therapy in patients with SSc overall points in the direction of beneficial effects and no adverse outcomes, but the evidence is weak. The conclusion of this review is in line with three reviews conducted in the past 4 years [10, 22, 29], one being a systematic literature review [10]. Previous literature reviews concluded that SSc patients without or with mild pulmonary involvement can be as physically active as the general population, whereas the present review provides more insights into the effect of exercise therapy on top of other non-pharmacological interventions, into different types of exercise programmes and into the methodological quality of the studies.

The present study makes it all the more clear that research in this area is very scanty, and the available evidence is weak. The weakness of the evidence is mainly attributable to the lack of studies with a randomized, controlled design. In addition, probably related to the fact that SSc is a relatively rare disease, the sample sizes were generally small. It should also be noted that the aims and contents of exercise programmes varied greatly, for which purpose we categorized the programmes into hand exercises, orofacial exercises and aerobic and muscle-strengthening exercises. Besides, the outcome parameters used differed greatly between the studies, making it difficult to judge the generalizability of the shown efficacy.

Moreover, it is important to note that the interventions in the studies on aerobic exercise in the present systematic review [12, 23, 27, 42, 43] did not meet public health recommendations for health-enhancing physical activity. The total duration of supervised moderate-intensity exercise ranged from 30 to 50 min, whereas for healthy adults the American College of Sports Medicine recommends to undertake ≥ 150 to 300 min of moderate-intensity exercise. Such recommendations are advocated for all patients with rheumatic and musculoskeletal diseases [35]. Of course, it is natural that the included interventions did not meet the public health recommendations because they focused on a single aspect of exercise; however, we believe that more attention to the minimum requirements of exercise to attain health benefits is needed.

In seven of the nine studies, no adverse events related to the exercise programmes were reported [12, 23, 27, 41–44]. Only one study [11] reported muscular fatigue as an adverse event, which occurred during the exercise programme and disappeared within 30 min after finishing the exercises. However, the quality assessment revealed that in most of the cases the reporting of potential adverse events was not part of the outcome assessment. Therefore, adverse events might be under-reported. It is likely that the systematic recording of potential adverse effects of exercise therapy in clinical studies needs more attention. This could be achieved by including it in checklists, such as those of The CONSORT group. Currently, in the CONSORT statement on the reporting of research on non-pharmacological treatment with the description of outcomes, potential adverse events are not particularly mentioned [52]. Although the current evidence suggests that physical exercise in SSc patients appears to be safe, it is important to screen patients beforehand and for exercise to be supervised by qualified health professionals, especially if patients have cardiopulmonary involvement. As an example, in an RCT on a multidisciplinary intervention including exercise therapy, potentially eligible patients with SSc had to undergo screening tests to assess their exercise intolerance, with the results being discussed by a cardiologist and a pulmonologist [19]. In other rheumatic conditions, such as OA, protocols on how to adapt exercise to co-morbidity have been developed [53].

Additionally, when assessing the methodological quality, none of the included studies had the maximum score for internal validity. The maximum score for internal validity was 13 points, based on seven questions concerning bias and six concerning confounding. The score for internal validity ranged from three to eight points. Only four of the nine studies scored more than half of the 13 points. This indicates methodological shortcomings of the included studies. For future exercise interventions, it is important to bear this in mind when designing the intervention.

Limitations of our study are that a meta-analysis was not performed because of the variety of interventions and outcomes and that we focused on exercise therapy, implying involvement of a health professional, as opposed to fully unsupervised home exercise or the promotion of physical activity. The search strategy we used was very broad, but did not yield studies on unsupervised exercise or physical activity. Furthermore, we included only eight studies, with small study populations, which makes it hard to draw firm conclusions. Another limitation of our study was that we extracted data only on outcomes directly related to exercise therapy, i.e. physical functioning, and not on outcomes other than physical functioning, such as appearance/body image or mouth hygiene.

## Conclusion

In conclusion, the literature on the effect of exercise therapy in patients with SSc is scanty and diverse. Overall, exercise therapy is described as safe, and studies indicate a possible positive effect, but no firm conclusions can be drawn. Given the high variability and the fact that some studies show that possible effects and adherence might wane after stopping the programme, it is important to evaluate specific preferences and needs of the patient. Collaborative efforts to conduct methodologically sound intervention studies are needed, taking into account specific disease-related factors, such as lung involvement and fatigue, and with adequate reporting.

*Funding:* No specific funding was received from any funding bodies in the public, commercial or not-for-profit sectors to carry out the work described in this manuscript.

*Disclosure statement*: The authors have declared no conflicts of interest.

## Supplementary Material

rkz044_Supplementary_Data

## References

[1] Hinchcliff M, Varga J. Systemic sclerosis/scleroderma: a treatable multisystem disease. Am Fam Physician 2008;78:961–8. 18953973

[2] Bassel M, Hudson M, Taillefer SS et al Frequency and impact of symptoms experienced by patients with systemic sclerosis: results from a Canadian National Survey. Rheumatology 2011;50:762–7. 21149249 10.1093/rheumatology/keq310

[3] Cuomo G, Santoriello C, Polverino F et al Impaired exercise performance in systemic sclerosis and its clinical correlations. Scand J Rheumatol 2010;39:330–5. 20476863 10.3109/03009740903555358

[4] Pettersson H, Åkerström A, Nordin A et al Self-reported physical capacity and activity in patients with systemic sclerosis and matched controls. Scand J Rheumatol 2017;46:490–5. 28303747 10.1080/03009742.2017.1281436

[5] Liem SIE, Meessen J, Wolterbeek R et al Physical activity in patients with systemic sclerosis. Rheumatol Int 2018;38:443–53. 29151128 10.1007/s00296-017-3879-y PMC5847038

[6] Kowal-Bielecka O, Landewe R, Avouac J et al EULAR recommendations for the treatment of systemic sclerosis: a report from the EULAR Scleroderma Trials and Research group (EUSTAR). Ann Rheum Dis 2009;68:620–8. 19147617 10.1136/ard.2008.096677

[7] Smidt N, de Vet HC, Bouter LM et al Effectiveness of exercise therapy: a best-evidence summary of systematic reviews. Aust J Physiother 2005;51:71–85. 15924510 10.1016/s0004-9514(05)70036-2

[8] Meijs J, Zirkzee EJ, Schouffoer AA et al Health-care utilization in Dutch systemic sclerosis patients. Clin Rheumatol 2014;33:825–32. 23982563 10.1007/s10067-013-2373-5

[9] Willems LM, Kwakkenbos L, Bode C, van den Hoogen FH, van den Ende CH. Health care use and patients' perceptions on quality of care in systemic sclerosis. Clin Exp Rheumatol 2013;31(2 Suppl 76):64–70. 23910612

[10] Willems LM, Vriezekolk JE, Schouffoer AA et al Effectiveness of nonpharmacologic interventions in systemic sclerosis: a systematic review. Arthritis Care Res 2015;67:1426–39. 10.1002/acr.22595 25832447

[11] Pizzo G, Scardina GA, Messina P. Effects of a nonsurgical exercise program on the decreased mouth opening in patients with systemic scleroderma. Clin Oral Investig 2003;7:175–8. 10.1007/s00784-003-0216-5 14513305

[12] Pinto AL, Oliveira NC, Gualano B et al Efficacy and safety of concurrent training in systemic sclerosis. J Strength Cond Res 2011;25:1423–8. 21116202 10.1519/JSC.0b013e3181d6858b

[13] Yuen HK, Marlow NM, Reed SG et al Effect of orofacial exercises on oral aperture in adults with systemic sclerosis. Disabil Rehabil 2012;34:84–9. 21951278 10.3109/09638288.2011.587589 PMC3437654

[14] Sandqvist G, Åkesson A, Eklund M. Evaluation of paraffin bath treatment in patients with systemic sclerosis. Disabil Rehabil 2004;26:981–7. 15371046 10.1080/09638280410001702405

[15] Antonioli CM, Bua G, Frigè A et al An individualized rehabilitation program in patients with systemic sclerosis may improve quality of life and hand mobility. Clin Rheumatol 2009;28:159–65. 18795394 10.1007/s10067-008-1006-x

[16] Maddali Bongi S, Del Rosso A, Galluccio F et al Efficacy of a tailored rehabilitation program for systemic sclerosis. Clin Exp Rheumatol 2009;27(3 Suppl 54):44–50. 19796561

[17] Bongi SM, Del Rosso A, Galluccio F et al Efficacy of connective tissue massage and Mc Mennell joint manipulation in the rehabilitative treatment of the hands in systemic sclerosis. Clin Rheumatol 2009;28:1167–73. 19554274 10.1007/s10067-009-1216-x

[18] Maddali-Bongi S, Landi G, Galluccio F et al The rehabilitation of facial involvement in systemic sclerosis: efficacy of the combination of connective tissue massage, Kabat's technique and kinesitherapy: a randomized controlled trial. Rheumatol Int 2011;31:895–901. 20238221 10.1007/s00296-010-1382-9

[19] Schouffoer AA, Ninaber MK, Beaart-van de Voorde LJ et al Randomized comparison of a multidisciplinary team care program with usual care in patients with systemic sclerosis. Arthritis Care Res 2011;63:909–17. 10.1002/acr.20448 21312348

[20] Poole JL, Skipper B, Mendelson C. Evaluation of a mail-delivered, print-format, self-management program for persons with systemic sclerosis. Clin Rheumatol 2013;32:1393–8. 23652719 10.1007/s10067-013-2282-7

[21] Poole JL, Mendelson C, Skipper B, Khanna D. Taking charge of systemic sclerosis: a pilot study to assess the effectiveness of an internet self-management program. Arthritis Care Res 2014;66:778–82. 10.1002/acr.22192 24115761

[22] de Oliveira NC, Portes LA, Pettersson H, Alexanderson H, Boström C. Aerobic and resistance exercise in systemic sclerosis: state of the art. Musculoskeletal Care 2017;15:316–23. 28378937 10.1002/msc.1185

[23] Oliveira NC, dos Santos Sabbag LM, de Sa Pinto AL, Borges CL, Lima FR. Aerobic exercise is safe and effective in systemic sclerosis. Int J Sports Med 2009;30:728–32. 19642060 10.1055/s-0029-1224180

[24] Shoemaker MJ, Wilt JL, Dasgupta R, Oudiz RJ. Exercise training in patients with pulmonary arterial hypertension: a case report. Cardiopulm Phys Ther J 2009;20:12–8. 20467524 PMC2845256

[25] Chernev I, Gustafson K, Medina-Bravo A. Functional outcome in a patient with an acute quadriparesis secondary to systemic sclerosis: a case report. Arch Phys Med Rehabil 2009;90:170–2. 19154843 10.1016/j.apmr.2008.06.033

[26] Mugii N, Someya F, Hasegawa M. Reduced hypoxia risk in a systemic sclerosis patient with interstitial lung disease after long-term pulmonary rehabilitation. Clin Med Insights Case Rep 2011;4:53–6. 22084615 10.4137/CCRep.S8071 PMC3210624

[27] Alexanderson H, Bergegård J, Björnådal L, Nordin A. Intensive aerobic and muscle endurance exercise in patients with systemic sclerosis: a pilot study. BMC Res Notes 2014;7:86. 24507585 10.1186/1756-0500-7-86 PMC3996139

[28] Rannou F, Boutron I, Mouthon L et al Personalized physical therapy versus usual care for patients with systemic sclerosis: a randomized controlled trial. Arthritis Care Res 2017;69:1050–9. 10.1002/acr.23098 27696703

[29] Mugii N, Hamaguchi Y, Maddali-Bongi S. Clinical significance and usefulness of rehabilitation for systemic sclerosis. J Scleroderma Relat Disord 2018;3:71–80. 10.1177/2397198317750043 PMC8892873 35382125

[30] Mugii N, Hasegawa M, Matsushita T et al The efficacy of self-administered stretching for finger joint motion in Japanese patients with systemic sclerosis. J Rheumatol 2006;33:1586–92. 16881115

[31] Bongi SM, Del Rosso A, Passalacqua M, Miccio S, Cerinic MM. Manual lymph drainage improving upper extremity edema and hand function in patients with systemic sclerosis in edematous phase. Arthritis Care Res 2011;63:1134–41. 10.1002/acr.20487 21523925

[32] Someya F, Mugii N. Pulmonary rehabilitation outcome of exercise-induced oxygen desaturation in systemic sclerosis with interstitial lung disease. J Health 2013; 05:1–5. 10.4187/respcare.02452 23764864

[33] Moher D, Liberati A, Tetzlaff J, Altman DG. Preferred reporting items for systematic reviews and meta-analyses: the PRISMA statement. PLoS Med 2009;6:e1000097. 19621072 10.1371/journal.pmed.1000097 PMC2707599

[34] Shea BJ, Reeves BC, Wells G et al AMSTAR 2: a critical appraisal tool for systematic reviews that include randomised or non-randomised studies of healthcare interventions, or both. BMJ 2017;358:j4008. 28935701 10.1136/bmj.j4008 PMC5833365

[35] Rausch Osthoff AK, Niedermann K, Braun J et al 2018 EULAR recommendations for physical activity in people with inflammatory arthritis and osteoarthritis. Ann Rheum Dis 2018;77:1251–60. 29997112 10.1136/annrheumdis-2018-213585

[36] Furlan AD, Pennick V, Bombardier C, van Tulder M. 2009 updated method guidelines for systematic reviews in the Cochrane Back Review Group. Spine 2009;34:1929–41. 19680101 10.1097/BRS.0b013e3181b1c99f

[37] Downs SH, Black N. The feasibility of creating a checklist for the assessment of the methodological quality both of randomised and non-randomised studies of health care interventions. J Epidemiol Commun Health 1998;52:377–84. 10.1136/jech.52.6.377 PMC1756728 9764259

[38] Ghogomu EA, Maxwell LJ, Buchbinder R et al Updated method guidelines for Cochrane musculoskeletal group systematic reviews and metaanalyses. J Rheumatol 2014;41:194–205. 24293581 10.3899/jrheum.121306

[39] Preliminary criteria for the classification of systemic sclerosis (scleroderma). Subcommittee for scleroderma criteria of the American Rheumatism Association diagnostic and therapeutic criteria committee. Arthritis Rheum 1980;23:581–90. 7378088 10.1002/art.1780230510

[40] van den Hoogen F, Khanna D, Fransen J et al 2013 classification criteria for systemic sclerosis: an American College of Rheumatology/European League against Rheumatism collaborative initiative. Arthritis Rheum 2013;65:2737–47. 24122180 10.1002/art.38098 PMC3930146

[41] Landim SF, Bertolo MB, Marcatto de Abreu MF et al The evaluation of a home-based program for hands in patients with systemic sclerosis. J Hand Ther 2019;32:313–21. 29198478 10.1016/j.jht.2017.10.013

[42] Mitropoulos A, Gumber A, Crank H, Akil M, Klonizakis M. The effects of upper and lower limb exercise on the microvascular reactivity in limited cutaneous systemic sclerosis patients. Arthritis Res Ther 2018;20:112. 29871697 10.1186/s13075-018-1605-0 PMC5989435

[43] Mitropoulos A, Gumber A, Akil M, Klonizakis M. Exploring the microcirculatory effects of an exercise programme including aerobic and resistance training in people with limited cutaneous systemic sclerosis. Microvascular Res 2019;125:103887. 10.1016/j.mvr.2019.103887 31220505

[44] Piga M, Tradori I, Pani D et al Telemedicine applied to kinesiotherapy for hand dysfunction in patients with systemic sclerosis and rheumatoid arthritis: recovery of movement and telemonitoring technology. J Rheumatol 2014;41:1324–33. 24882841 10.3899/jrheum.130912

[45] Mitropoulos A, Gumber A, Crank H, Akil M, Klonizakis M. Investigating the effectiveness and feasibility of exercise on microvascular reactivity and quality of life in systemic sclerosis patients: study protocol for a feasibility study. Trials 2018;19:647. 30463598 10.1186/s13063-018-2980-1 PMC6249907

[46] Sydow E, Arat S, Nijs G et al An explorative study evaluating feasibility and effectiveness of two different exercise programs in systemic sclerosis associated microstomia. J Scleroderma Relat Disord 2018;3:199–209.

[47] Sydow E, Severi S, Van der Elst K et al A mixed method study to explore the feasibility and patient satisfaction of two different exercise programs in systemic sclerosisassociated microstomia. Ann Rheum Dis 2019;78:1060.

[48] Kwakkenbos L, Carrier ME, Boutron I et al Randomized feasibility trial of the scleroderma patient-centered intervention network hand exercise program (SPIN-HAND). J Scleroderma Relat Disord 2018;3:91–97. 10.5301/jsrd.5000263 PMC8892877 35382119

[49] NCT03092024. Scleroderma Patient-Centered Intervention Network (SPIN) Hand Program Feasibility Study. https://clinicaltrials.gov/ct2/show/nct03092024 (Last accessed November 27, 2019).

[50] NCT03419208. Scleroderma Patient-Centered Intervention Network (SPIN) Hand Program. https://clinicaltrials.gov/ct2/show/NCT03419208 (last accessed November 27, 2019).

[51] NCT03614208. Individualized Home-Based Exercise Program for Patient with Systemic Sclerosis. https://clinicaltrials.gov/ct2/show/nct03614208 (Last accessed November 27, 2019).

[52] Boutron I, Altman DG, Moher D, Schulz KF, Ravaud P. CONSORT statement for randomized trials of nonpharmacologic treatments: a 2017 update and a CONSORT extension for nonpharmacologic trial abstracts. Ann Intern Med 2017;167:40–7. 28630973 10.7326/M17-0046

[53] de Rooij M, van der Leeden M, Avezaat E et al Development of comorbidity-adapted exercise protocols for patients with knee osteoarthritis. Clin Interv Aging 2014;9:829–42. 24868151 10.2147/CIA.S55705 PMC4027930

