# Supplementary material for: The effect and safety of exercise therapy in patients with systemic sclerosis: a systematic review
Source: Rheumatol Adv Pract. 2019 Dec 9;3(2):rkz044. doi: 10.1093/rap/rkz044 (PMC6913710; doi:10.1093/rap/rkz044)
Supplement: rkz044_Supplementary_Data [file rkz044_supplementary_data.docx]

**SUPPLEMENTARY MATERIAL**

**Search strategy**

This search strategy was performed on September 3^rd^ 2019 by JWS, a trained librarian.

(("Scleroderma, Systemic"[Mesh] OR "systemic sclerosis"[tw] OR "systemic scleroderma"[tw] OR systemic sclero*[tw] OR systemic sclero*[tw] OR "diffuse scleroderma"[tw] OR "limited scleroderma"[tw] OR "CREST Syndrome"[tw] OR "CRST Syndrome"[tw] OR "scleroderma"[tw]) AND (exercis*[tw] OR "stretching"[tw] OR "Exercise Therapy"[Mesh] OR "exercise therapy"[tw] OR exercise therap*[tw] OR "Muscle Stretching Exercises"[tw] OR "Muscle Stretching Exercise"[tw] OR "Static Stretching"[tw] OR "Passive Stretching"[tw] OR "Static-Passive Stretching"[tw] OR "Static Passive Stretching"[tw] OR "Isometric Stretching"[tw] OR "Active Stretching"[tw] OR "Static-Active Stretching"[tw] OR "Static Active Stretching"[tw] OR "Ballistic Stretching"[tw] OR "Dynamic Stretching"[tw] OR "PNF Stretching"[tw] OR "Plyometric Exercise"[tw] OR "Plyometric Exercises"[tw] OR Plyometric Drill*[tw] OR "Plyometric Drills"[tw] OR "Plyometric Training"[tw] OR "Plyometric Trainings"[tw] OR "Stretch-Shortening Exercise"[tw] OR "Stretch Shortening Exercise"[tw] OR "Stretch-Shortening Exercises"[tw] OR "Stretch-Shortening"[tw] OR "Stretch Shortening"[tw] OR "Stretch-Shortening Drills"[tw] OR "Stretch-Shortening Cycle Exercise"[tw] OR "Stretch Shortening Cycle Exercise"[tw] OR "Stretch-Shortening Cycle Exercises"[tw] OR "Resistance Training"[tw] OR "Strength Training"[tw] OR "Weight-Bearing"[tw] OR "Weight Bearing"[tw] OR "Exercise"[Mesh] OR "Exercise"[tw] OR "Exercises"[tw] OR "Physical Exercise"[tw] OR "Physical Exercises"[tw] OR "Isometric Exercises"[tw] OR "Isometric Exercise"[tw] OR "Aerobic Exercises"[tw] OR "Aerobic Exercise"[tw] OR "Circuit-Based Exercise"[tw] OR "Cool-Down Exercise"[tw] OR "Cool-Down Exercises"[tw] OR "Physical Conditioning"[tw] OR "Running"[tw] OR "Jogging"[tw] OR "Swimming"[tw] OR "Walking"[tw] OR "Warm-Up Exercise"[tw] OR "Warm-Up Exercises"[tw] OR "Physical Exertion"[Mesh] OR "Physical Exertion"[tw] OR "Physical Effort"[tw] OR "Physical Efforts"[tw] OR "Physical Fitness"[Mesh] OR "Physical Fitness"[tw] OR "Physical Endurance"[mesh] OR "Physical Endurance"[tw] OR "Anaerobic Threshold"[tw] OR "Exercise Tolerance"[tw] OR "Exercise Movement Techniques"[Mesh] OR "Exercise Movement"[tw] OR "Sports"[Mesh] OR "Sport"[tw] OR "Sports"[tw] OR "Walking"[tw] OR "Motor Activity"[Mesh] OR "Physical Activity"[tw] OR exertion*[tw] OR treadmill*[tw] OR swim*[tw] OR bicycl*[tw] OR cycling[tw] OR walk*[tw] OR muscle strength*[tw]) NOT ("Animals"[mesh] NOT "Humans"[mesh])) AND ("1990/01/01"[PDAT] : "3000/12/31"[PDAT])

**Modified Downs and Black Checklist for evaluating study quality**

| **Quality items** | **Specification of items** |
| --- | --- |
| **Reporting** |  |
| 1. Is the hypothesis/aim/objective of the study clearly described?   \| yes \| 1 \| \| --- \| --- \| \| no \| 0 \| | Studies must report on what outcome domain(s) the intervention aims to be effective. |
| 2. Are the (main) outcomes to be measured clearly described in the Introduction or Methods section?   \| yes \| 1 \| \| --- \| --- \| \| no \| 0 \| | If the main outcomes are first mentioned in the Results section, the question should be answered no. |
| 3. Are the characteristics of the patients included in the study clearly described?   \| yes \| 1 \| \| --- \| --- \| \| no \| 0 \| | In cohort studies and trials, inclusion and/or exclusion criteria should be given. In case-control studies, a case-definition and the source for controls should be given. |
| 4. Are the interventions of interest clearly described?   \| yes \| 1 \| \| --- \| --- \| \| no \| 0 \| | Treatments and placebo (where relevant) that are to be compared should be clearly described (including duration and frequency). |
| 5. Are the distributions of principal confounders in each group of subjects to be compared clearly described?   \| yes \| 2 \| \| --- \| --- \| \| partially \| 1 \| \| no \| 0 \| | A list of principal confounders is provided. In the Methods section it must be explicitly mentioned that confounders were taken into account. When baseline comparison between groups are present, this should be answered partially. |
| 6. Are the (main) findings of the study clearly described?   \| yes \| 1 \| \| --- \| --- \| \| no \| 0 \| | Simple outcome data (including denominators and numerators) should be reported for all major findings so that the reader can check the major analyses and conclusions. (This question does not cover statistical tests which are considered below). |
| 7. Does the study provide estimates of the random variability in the data for the main outcomes?   \| yes \| 1 \| \| --- \| --- \| \| no \| 0 \| | In non-normally distributed data the inter-quartile range of results should be reported. In normally distributed data the standard error, standard deviation or confidence intervals should be reported. If the distribution of the data is not described, it must be assumed that the estimates used were appropriate and the question should be answered yes. If not applicable, this should be answered no. |
| 8. Have all important adverse events that may be a consequence of the intervention been reported?   \| yes \| 1 \| \| --- \| --- \| \| no \| 0 \| | In the Methods section it must be mentioned that adverse events were assessed. |
| 9. Have the characteristics of patients lost to follow-up been described?   \| yes \| 1 \| \| --- \| --- \| \| no \| 0 \| | This should be answered yes where there were no losses to follow-up or where losses to follow-up were so small that findings would be unaffected by their inclusion. This should be answered no where a study does not report the number of patients lost to follow-up. |
| 10. Have actual probability values been reported (e.g. 0.035 rather than <0.05) for the (main) outcomes except where the probability value is less than 0.001?   \| yes \| 1 \| \| --- \| --- \| \| no \| 0 \| | In table or text. |
| **External validity** |  |
| 11. Were the subjects asked to participate in the study representative of the entire population from which they were recruited?   \| yes \| 1 \| \| --- \| --- \| \| no \| 0 \| \| unable to determine \| 0 \| | The study must identify the source population for patients and describe how the patients were selected. Patients would be representative if they comprised the entire source population, an unselected sample of consecutive patients, or a random sample. Random sampling is only feasible where a list of all members of the relevant population exists. Where a study does not report the proportion of the source population from which the patients are derived, the question should be answered as unable to determine. |
| 12. Were those subjects who were prepared to participate representative of the entire population from which they were recruited?   \| yes \| 1 \| \| --- \| --- \| \| no \| 0 \| \| unable to determine \| 0 \| | The proportion of those asked who agreed should be stated. Validation that the sample was representative would include demonstrating that the distribution of the main confounding factors was the same in the study sample and the source population. |
| 13. Were the staff, places, and facilities where the patients were treated, representative of the treatment the majority of patients receive?   \| yes \| 1 \| \| --- \| --- \| \| no \| 0 \| \| unable to determine \| 0 \| | The question should be answered no if, for example, the intervention was undertaken in a specialist centre unrepresentative of the hospitals most of the source population would attend. |
| **Internal validity- bias** |  |
| 14. Was an attempt made to blind study subjects to the intervention they have received?   \| yes \| 1 \| \| --- \| --- \| \| no \| 0 \| \| unable to determine \| 0 \| | For studies where the patients would have no way of knowing which intervention they received, this should be answered yes. Cohort/ observational studies should be answered no. |
| 15. Was an attempt made to blind those measuring the (main) outcomes of the intervention?   \| yes \| 1 \| \| --- \| --- \| \| no \| 0 \| \| unable to determine \| 0 \| | Cohort/ observational studies should be answered no. |
| 16. If any of the results of the study were based on “data dredging”, was this made clear?   \| yes \| 1 \| \| --- \| --- \| \| no \| 0 \| \| unable to determine \| 0 \| | Any outcome analyses that had not been planned at the outset of the study should be clearly indicated. If no retrospective unplanned subgroup analyses were reported, then answer yes. |
| 17. In trials and cohort studies, do the analyses adjust for different lengths of follow-up of patients, or in case-control studies, is the time period between the intervention and outcome the same for cases and controls?   \| yes \| 1 \| \| --- \| --- \| \| no \| 0 \| \| unable to determine \| 0 \| | Where follow-up was the same for all study patients the answer should be yes. If different lengths of follow-up were adjusted for by, for example, survival analysis the answer should be yes. Studies where differences in follow-up are ignored should be answered no. |
| 18. Were the statistical tests used to assess the (main) outcomes appropriate?   \| yes \| 1 \| \| --- \| --- \| \| no \| 0 \| \| unable to determine \| 0 \| | The statistical techniques used must be appropriate to the data. For example non-parametric methods should be used for small sample sizes. Where little statistical analysis has been undertaken but where there was no evidence of bias, the question should be answered yes. If the distribution of the data (normal or not) is not described it must be assumed that the estimates used were appropriate and the question should be answered yes. |
| 19. Was compliance with the intervention/s reliable?   \| yes \| 1 \| \| --- \| --- \| \| no \| 0 \| \| unable to determine \| 0 \| | Where there was non-compliance with the allocated treatment or where there was contamination of one group, the question should be answered no. For studies where the effect of any misclassification was likely to bias any association to the null, the question should be answered yes. |
| 20. Were the (main) outcome measures used accurate (valid and reliable)?   \| yes \| 1 \| \| --- \| --- \| \| no \| 0 \| \| unable to determine \| 0 \| | For studies where the outcome measures are clearly described, the question should be answered yes. For studies which refer to other work or that demonstrates the outcome measures are accurate or widely accepted in rheumatic diseases, the question should be answered yes. |
| **Internal validity- confounding** |  |
| 21. Were the patients in different intervention groups (trials and cohort studies) or were the cases and controls (case-control studies) recruited from the same population?   \| yes \| 1 \| \| --- \| --- \| \| no \| 0 \| \| unable to determine \| 0 \| | For example, patients for all comparison groups should be selected from the same hospital. The question should be answered unable to determine for cohort and case-control studies where there is no information concerning the source of patients included in the study. Cohort/ observational studies should be answered no. |
| 22. Were study subjects in different intervention groups (trials and cohort studies) or were the cases and controls (case-control studies) recruited over the same period of time?   \| yes \| 1 \| \| --- \| --- \| \| no \| 0 \| \| unable to determine \| 0 \| | For the study which does not specify the time period over which patients were recruited, the question should be answered as unable to determine. |
| 23. Were study subjects randomised to intervention groups?   \| yes \| 1 \| \| --- \| --- \| \| no \| 0 \| \| unable to determine \| 0 \| | Studies which state that subjects were randomised should be answered yes except where method of randomisation would not ensure random allocation. For example alternate allocation would score no because it is predictable. |
| 24. Was the randomised intervention assignment concealed from both patients and health care staff until recruitment was complete and irrevocable?   \| yes \| 1 \| \| --- \| --- \| \| no \| 0 \| \| unable to determine \| 0 \| | All non-randomised studies should be answered no. If assignment was concealed from patients but not from staff, it should be answered no. |
| 25. Was there adequate adjustment for confounding in the analyses from which the (main) findings were drawn?   \| yes \| 1 \| \| --- \| --- \| \| no \| 0 \| \| unable to determine \| 0 \| | This question should be answered no for trials if: the main conclusions of the study were based on analyses of treatment rather than intention to treat; the distribution of known confounders in the different treatment groups was not described; or the distribution of known confounders differed between the treatment groups but was not taken into account in the analyses. In non-randomised studies if the effect of the main confounders was not investigated or confounding was demonstrated but no adjustment was made in the final analyses the question should be answered as no. |
| 26. Were losses of patients to follow-up taken into account?   \| yes \| 1 \| \| --- \| --- \| \| no \| 0 \| \| unable to determine \| 0 \| | If the numbers of patients lost to follow-up are not reported, the question should be answered as unable to determine. If the proportion lost to follow-up was too small to affect the main findings, the question should be answered yes (< 20% lost to follow-up). |
| **Power** |  |
| 27. Did the study included a power analysis?   \| yes \| 1 \| \| --- \| --- \| \| no \| 0 \| | When a power analysis is conducted this question should be answered yes. |
